# AI-based fully automatic image analysis: Optimal abdominal and thoracic segmentation volumes for estimating total muscle volume on computed tomography scans

**DOI:** 10.1016/j.afos.2024.04.001

**Published:** 2024-04-24

**Authors:** Thomas Ying, Pablo Borrelli, Lars Edenbrandt, Olof Enqvist, Reza Kaboteh, Elin Trägårdh, Johannes Ulén, Henrik Kjölhede

**Affiliations:** a Department of Urology, Sahlgrenska University Hospital, Blå Stråket 5, 41345, Gothenburg, Sweden; b Department of Urology, Institute of Clinical Sciences, Sahlgrenska Academy, University of Gothenburg, Medicinaregatan 3, 40530, Gothenburg, Sweden; c Department of Clinical Physiology, Sahlgrenska University Hospital, Blå Stråket 5, 41345, Gothenburg, Sweden; d Department of Molecular and Clinical Medicine, Institute of Medicine, Sahlgrenska Academy, University of Gothenburg, Medicinaregatan 3, 40530, Gothenburg, Sweden; e Eigenvision AB, Bredgatan 4, 21130, Malmö, Sweden; f Department of Translational Medicine and Wallenberg Centre for Molecular Medicine, Lund University, Margaretavägen 1 A, 22240, Lund, Sweden; g Clinical Physiology and Nuclear Medicine, Skåne University Hospital, Carl-Bertil Laurells Gata 9, 21428, Malmö, Sweden

**Keywords:** Image analysis (computer-assisted), Body composition, Sarcopenia, Artificial intelligence

## Abstract

**Objectives:**

Evaluation of sarcopenia from computed tomography (CT) is often based on measuring skeletal muscle area on a single transverse slice. Automatic segmentation of muscle volume has a lower variance and may be a better proxy for the total muscle volume than single-slice areas. The aim of the study was to determine which abdominal and thoracic anatomical volumes were best at predicting the total muscle volume.

**Methods:**

A cloud-based artificial intelligence tool (recomia.org) was used to segment all skeletal muscle of the torso of 994 patients who had performed whole-torso CT 2008–2020 for various clinical indications. Linear regression models for several anatomical volumes and single-slice areas were compared with regard to predicting the total torso muscle volume.

**Results:**

The muscle volume from the tip of the coccyx and 25 cm cranially was the best of the abdominal volumes and was significantly better than the L3 slice muscle area (R^2^ 0.935 vs 0.830, P < 0.0001). For thoracic volumes, the muscle volume between the top of the sternum to the lower bound of the Th12 vertebra showed the best correlation with the total volume, significantly better than the Th12 slice muscle area (R^2^ 0.892 vs 0.775, P < 0.0001). Adjusting for body height improved the correlation slightly for all measurements but did not significantly change the ordering.

**Conclusions:**

We identified muscle volumes that can be reliably segmented by automated image analysis which is superior to single slice areas in predicting total muscle volume.

## 1 Introduction

Sarcopenia, ie, reduction of muscle mass with loss of muscle function, is associated with an increased risk of postoperative complications and shorter survival in several types of cancer surgery [[1], [2], [3], [4]]. One of the ways to identify sarcopenia is to use computed tomography (CT) examinations, where the most common method is to use the muscle area at a cross-section of lumbar vertebra 3 (L3) or thoracic vertebra 12 (Th12), for which cut-off thresholds have been proposed [[5], [6], [7], [8], [9]]. This has generally been done manually by radiologists in the studies. However, due to resource and time consumption, it has rarely been established in clinical use. Moreover, the relationship between one cross section and the total muscle mass carries significant uncertainty on an individual basis.

The development of Artificial Intelligence (AI) has opened possibilities to automatically calculate the muscle area on CT in a very safe way, both more time-efficient and requiring fewer personnel resources. Commercially available solutions exist that measure the cross section skeletal muscle area [[10], [11], [12], [13]], but they still require some manual input and their clinical use have been limited. We have previously developed and described a cloud-based AI image analysis tool, the Research Consortium for Medical Image Analysis (RECOMIA). The website (recomia.org) can automatically measure skeletal muscle volume derived from CT scans and have shown that muscle volume has a lower variance than muscle area in L3 [14,15]. However, there is a lack of studies on which volume segments best correlate with the total muscle volume, and whether they are better than Th12/L3 as proxies of the total volume. Defining such optimal partial volumes would allow a better estimation of total muscle volume and sarcopenia from any CT scan of the abdomen or thorax. The goal of this study was to identify partial volumes, on CT of the thorax and of the abdomen, which correlate best with the total muscle volume and whether they perform better than Th12 or L3 slices.

## 2 Methods

### 2.1 *Patient background and study design*

The CT scans of the entire torso of patients who had undergone positron emission tomography/computed tomography (PET/CT) scans for various clinical indications at Sahlgrenska University Hospital in Gothenburg and Skåne University Hospital in Lund/Malmö from 2008 to 2020 were uploaded to the cloud-based AI image analysis tool Recomia.org. The majority were patients with prostate cancer (60%). The rest had lymphoma (20%), neuroendocrine cancer (10%) and other (10%). All patients were of legal age (18 years) at the time of imaging. Patients whose arms were alongside the body were excluded from analysis. The PET/CT scanners used for obtaining the CT images were from GE Healthcare Systems (models Discovery 690 and Discovery MI) and Siemens (models Biograph 64, Biograph 128 and Biograph 128 Edge).

Using the CT examinations for evaluating AI-based image analysis was approved by the Swedish Ethics Review Authority (2016/417 and 2021-05734-02).

### 2.2 *Management of image material*

During image uploading, all identifying information was automatically stripped from the digital imaging and communications in medicine (DICOM) tags of the scans. Some non-identifying patient information such as sex, age, height, and weight were preserved and later retrieved from the DICOM tags. All analysis was performed on anonymized data. Segmentation of the skeletal muscle tissue was done automatically by the AI tool without any manual intervention. The AI has also been trained to identify all the specific anatomical landmarks used in this study without the need for human input. One of the authors (TY) performed a visual inspection of a random selection of 50 of the cases to ensure that the volume segmentation was adequate and correct. The segmentations were classified as either correct or not. The AI-based image analysis has previously been described in detail and takes about 1 min per patient on a high-end desktop computer [14].

### 2.3 *Selection of muscle volume*

The cranial limit of the segmentation was the top of the sternum. ‘Sacrum and Coccyx’ was automatically segmented at the same time as muscle and fat, the most inferior slice of the automatic segmentation of coccyx was selected as the caudal limit. The volume of all skeletal muscle tissue between these reference points formed the total torso muscle volume used as reference in this study.

The cross-sectional areas of Th12 and L3 were measured because these are often used as proxies for the total muscle volume. There is lack of standardization and consensus at which part of the vertebra the cross-sectional area should be measured, here we chose to measure the area at the “center of mass” of vertebra Th12 and L3, respectively.

The pre-specified muscle volumes for abdominal CT scans that were analyzed were the volume between the tip of the coccyx and 25 cm cranially (Sacrum 25 cm), and the volume between the tip of the coccyx and the cranial limit of the first lumbar vertebra (Sacrum–L1). For thoracic CT scans the volumes were between the cranial border of the sternum and 25 cm caudally (Sternum 25 cm), the cranial border of the sternum to the caudal limit of the 12th thoracic vertebra (Sternum–Th12), and the cranial limit of 10th thoracic vertebra to the caudal limit of the 12th thoracic vertebra (Th10–12). The 25 cm volumes (Sacrum 25 cm and Sternum 25 cm) were chosen based on our previous studies on abdominal and thoracic CT scans, where 25 cm from the respective level represented the largest volumes that were consistently represented in clinical CT scans. Sacrum–L1 and Sternum–Th12 were similarly chosen to maximize the evaluated volume, while also taking the build of each patient into account.

### 2.4 *Statistical analysis*

For each of the measured areas and volumes, univariable linear regression analysis was done to predict the total torso muscle volume. For each such model, the Akaike Information Criterion (AIC), root mean squared error (RMSE) and R^2^ were calculated and analyzed for goodness-of-fit. The regression analyses were first performed for all patients. In a second analysis, both univariable and multivariable, also including the body height and the interaction between the volume/area and body height was done for the patients who had body height data. To evaluate the resulting models for patients of different ages, the patients were divided into quartiles. Further, correlation analysis was performed for each area or volume with regard to the total torso muscle volume, which was compared using the method described by Hittner et al. [16].

Scatter plots of the predicted total torso muscle volumes compared to the actual measured volume for each regression model was done, as well as Bland-Altman diagrams [17] for visual comparison.

Mean and standard deviation (SD) was calculated for each descriptive continuous variable. All statistical analysis was done using R version 3.6.3 (R Foundation for Statistical Computing, Vienna, Austria) with the packages *tidyverse* 1.3.1 and *cocor* 1.1–3.

## 3 Results

**Fig. 1:**
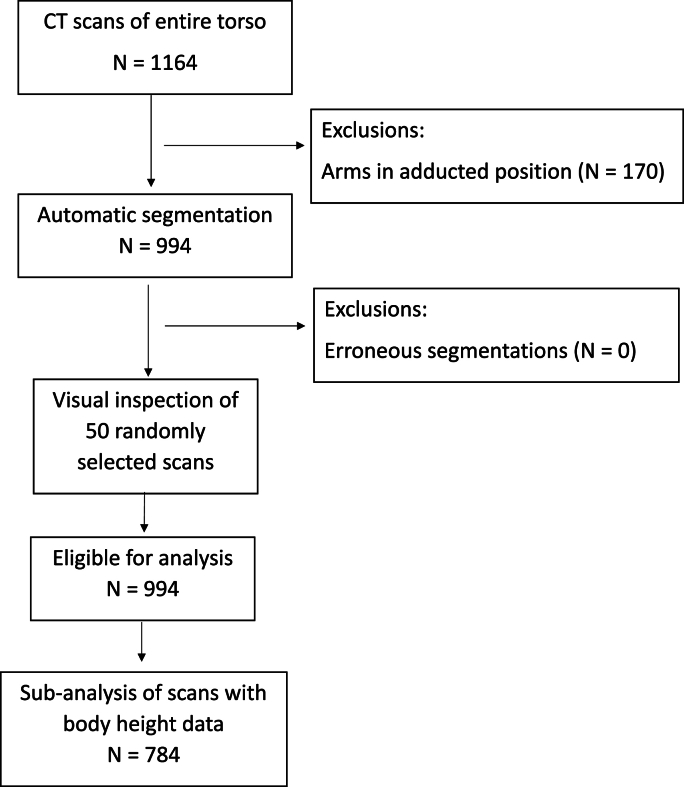
Flow chart of inclusion and exclusions of patients in the study.

A total of 1164 patients were included in the study. Of these, 170 were excluded due to the arms being held alongside the body, leaving 994 for analysis. Manual inspection of 50 randomly selected muscle segmentations did not show any that were obviously erroneous and did not lead to any exclusions (Fig. 1). The study group thus consisted of 821 (83%) men and 173 (17%) women, with a mean age of 64 years (SD 14) and a mean body length of 176 cm (SD 8) (Table 1). Intravenous contrast had been administered in 546 (55%) of the scans.

**Table 1:** Characteristics of the patients and their CT scans that were included in the study. Continuous variables are shown as mean (standard deviation), while categorical variables are shown as number of subjects (%).

Characteristic		Overall	No height	With height
		N = 994	N = 210	N = 784
Age, yrs		64 (14)	51 (17)	68 (10)
Female		173 (17%)	87 (41%)	86 (11%)
Male		821 (83%)	123 (59%)	698 (89%)
Height, m		1.76 (0.08)		1.76 (0.08)
Weight^a^, kg		83 (16)	78 (17)	85 (16)
BMI, kg/m^2^		27.2 (4.4)		27.2 (4.4)

Manufacturer				
SIEMENS		311 (31%)	206 (98%)	105 (13%)
GE Medical Systems		683 (69%)	4 (2%)	679 (87%)
kVp	80	8 (0.8%)	0 (0%)	8 (1.0%)
	100	325 (33%)	2 (1.0%)	323 (41%)
	120	661 (66%)	208 (99%)	453 (58%)
Slice Thickness, mm	2.5	197 (20%)	1 (0.5%)	196 (25%)
	3	312 (31%)	206 (98%)	106 (13%)
	5	485 (49%)	3 (1.4%)	482 (61%)
Intravenous contrast		546 (55%)	3 (1.4%)	543 (69%)

BMI, body mass index; kVp, kilovoltage peak.

a Weight were missing in 3 of the patients in the “No height” group.

On analyzing all 994 patients, Sacrum 25 cm showed the highest correlation of the abdominal measures on all analyses, with an R^2^ of 0.935 (Table 2). This was significantly higher than the L3 slice muscle area (R^2^ 0.830, P < 0.0001). For thoracic measures, Sternum–Th12 showed the highest correlation with total torso muscle volume, with an R^2^ of 0.892 which was significantly higher than the Th12 slice muscle area (R^2^ 0.775, P < 0.0001). The latter was marginally higher than Sternum 25 cm with an R^2^ of 0.891, but this difference was not statistically significant (P = 0.16).

**Table 2:** Performance characteristics of regression models for predicting total torso muscle volume from a limited volume or slice area. For the patients with body height data available, both regression models with and without height as covariate are shown. The best model in each category is shown in bold.

All patients (N = 994)				Patients with body height data (N = 784)			
	AIC	R^2^	RMSE		AIC	R^2^	RMSE
Abdomen							
Sacrum 25 cm	15,368	0.935	549	Sacrum 25 cm	12,151	0.922	559
				Sacrum 25 cm with height	12,052	0.932	524
Sacrum - L1	15,718	0.907	655	Sacrum - L1	12,445	0.887	675
				Sacrum - L1 with height	12,421	0.890	663
L3	16,321	0.830	887	L3	12,883	0.802	892
				L3 with height	12,716	0.840	800
Thorax							
Sternum 25 cm	15,882	0.891	711	Sternum 25 cm	12,557	0.869	725
				Sternum 25 cm with height	12,237	0.913	589
Sternum-Th12	15,875	0.892	709	Sternum-Th12	12,504	0.878	701
				Sternum-Th12 with height	12,313	0.905	619
Th10–Th12	16,440	0.809	942	Th10–Th12	12,986	0.774	953
				Th10–Th12 with height	12,872	0.805	883
Th12	16,603	0.775	1022	Th12	13,069	0.749	1004
				Th12 with height	12,817	0.819	853

AIC, Akaike information criterion; RMSE, root mean squared error; L3, level of the third lumbar vertebra; Th10, level of the 10th thoracic vertebra; Th12, level of the 12th thoracic vertebra.

**Fig. 2:**
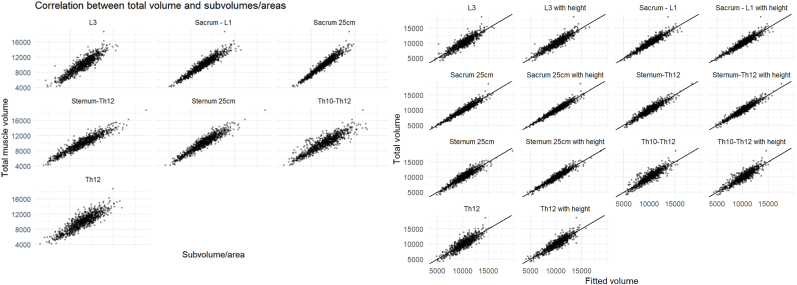
Scatterplots of the respective models against the total muscle volume. Darker areas show higher concentration of dots.

**Fig. 3:**
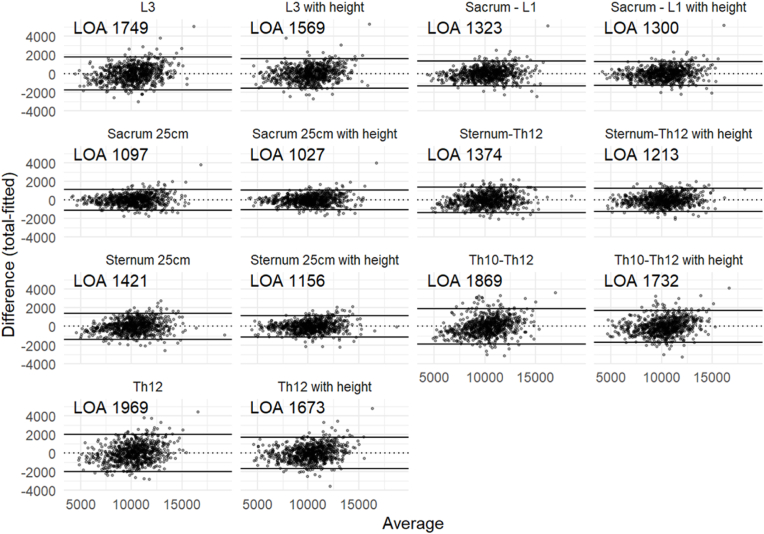
Bland-Altman plots of the respective models. Darker areas show higher concentration of dots. Limits of agreement (95% LOA) are shown for each model.

For the 784 patients where body length was available, for abdominal scans the multivariable model including Sacrum 25 cm improved only slightly (R^2^ 0.932 vs 0.922, P < 0.0001). In contrast, for thoracic scans, the multivariable model that included Sternum 25 cm improved markedly (R^2^ 0.913 vs 0.869, P < 0.0001) and became similar to the multivariable model that included Sternum–Th12 (R^2^ 0.913 vs 0.905, P = 0.05). A cross table of differences in correlations is presented in Table 3. All the evaluated volumes, except Th10–Th12, showed a significantly higher correlation with the total torso muscle volume than the single-slice areas, whether they were adjusted for body height or not (all P < 0.01; Supplemental Table 1). AIC and RMSE showed the same order of correlation as R^2^ for both univariable and multivariable models. Scatterplots of the respective measures against the total torso muscle volume visually demonstrate the same results (Fig. 2), as do the Bland-Altman plots (Fig. 3). Analyzing scans of male (N = 698) and female patients (N = 86) separately showed the same ordering of correlation of the models and yielded similar R^2^ in both groups (Sacrum 25 cm with height R^2^ 0.897 for men and 0.920 for women, P = 0.26 with Fisher's z method; Sternum 25 cm with height R^2^ 0.870 for men and 0.892 for women, P = 0.39). The parameters of all the regression models are shown in Supplemental Table 2. Evaluating the models for different age groups showed similar results for all age groups (Supplemental Table 3).

**Table 3:** Cross table of differences in correlations. Positive numbers mean that the column has a higher correlation than the row.

	L3	L3 with height	Sacrum - L1	Sacrum - L1 with height	Sacrum 25 cm	Sacrum 25 cm with height	Sternum-Th12	Sternum-Th12 with height	Sternum 25 cm	Sternum 25 cm with height	Th10–Th12	Th10–Th12 with height	Th12	Th12 with height
L3	–	0.021	0.046	0.048	0.065	0.070	0.041	0.055	0.037	0.060	−0.016	0.002	−0.030	0.009
L3 with height		–	0.025	0.027	0.043	0.048	0.020	0.034	0.015	0.039	−0.037	−0.019	−0.051	−0.012
Sacrum - L1			–	0.002	0.019	0.024	−0.005	0.009	−0.009	0.014	−0.062	−0.044	−0.076	−0.037
Sacrum - L1 with height				–	0.017	0.021	−0.007	0.007	−0.011	0.012	−0.064	−0.046	−0.078	−0.039
Sacrum 25 cm					–	0.005	−0.023	−0.009	−0.028	−0.004	−0.080	−0.062	−0.095	−0.055
Sacrum 25 cm with height						–	−0.028	−0.014	−0.033	−0.009	−0.085	−0.067	−0.100	−0.060
Sternum-Th12							–	0.014	−0.005	0.019	−0.057	−0.039	−0.071	−0.032
Sternum-Th12 with height								–	−0.019	0.005	−0.071	−0.053	−0.085	−0.046
Sternum 25 cm									–	0.023	−0.052	−0.035	−0.067	−0.027
Sternum 25 cm with height										–	−0.076	−0.058	−0.090	−0.051
Th10–Th12											–	0.018	−0.014	0.025
Th10–Th12 with height												–	−0.032	0.007
Th12													–	0.039
Th12 with height														–

## 4 Discussion

In this study, we found that the total torso muscle volume could be accurately estimated by automatic muscle segmentation of specific anatomically defined volumes on abdominal or thoracic CT scans, and that these volumes performed better than the commonly used single transverse cross-sectional muscle areas at L3 or Th12, whether adjusting for body length or not.

For abdominal CT scans, we found that the muscle volume between the tip of the coccyx and 25 cm cranially was the best at predicting the total torso muscle volume across all measures of goodness-of-fit. In their seminal paper, Shen and co-workers showed that the cross-sectional area at L3 was the best at predicting total muscle volume, with an R^2^ of 0.855. These results were later replicated by, eg, Schweitzer and co-workers, with an R^2^ of 0.76 in men but only 0.71 in women [7]. Similarly, we found an R^2^ of 0.830 for L3, although that was in relation to the total torso muscle volume, ie, not including the muscle volume of the extremities. While R^2^ is not directly comparable across studies, the similar results suggest that the improvement in prediction by measuring the Sacrum 25 cm volume rather than only a single cross section area is valid in general. In addition, we have previously reported that the pre-operative muscle volume in Sacrum 25 cm is an independent predictor of overall survival in patients who undergo radical cystectomy for urinary bladder cancer [1].

For thoracic CT scans, we found that the volume between the top of the sternum and the lower bound of the 1st lumbar vertebra was the best at predicting the total torso muscle value, although the top of the sternum and 25 cm caudally was essentially equally good. Both were significantly better than the single slice area at Th12, but also slightly worse than the best abdominal volume measure. To the best of our knowledge, no previous study has compared thoracic single-slice cross-sectional muscle areas to the total muscle volume, only comparisons to L3 slices or reference ranges in relation to L3 slices have been made [18,19]. Matsuyama and co-workers found a correlation coefficient of 0.804 between L3 and Th12 slices. We did not calculate the correlation between these slices but did find that Th12 had a worse correlation with the total torso muscle volume than L3. This agrees with the previously mentioned study by Shen and co-workers where a slice 15 cm above the L4-L5 disc was evaluated and which was worse than L3.

A recent meta-analysis has shown that lean muscle mass is a strong predictor of mortality in different forms of cancer [4]. This was also found in our previous report on mortality after radical cystectomy for bladder cancer [1]. Most studies, however, have used L3 single slices which we have shown here are less correlated to the total muscle volume than the partial volumes evaluated. Thereby, using automated segmentation of these volumes could allow a more accurate individual prediction of risks involved for the patients. However, cutoff values for sarcopenia will need to be established for these partial volumes [20].

The main strength of this study is the large number of whole-torso CT scans included. This was made possible by the automatic segmentation performed by cloud-based imaging analysis tool at Recomia.org. The main limitation is that we could not use total muscle volume as reference since the CT scans did not consistently include the extremities. Instead, we chose total torso muscle volume as a proxy, which may not entirely correlate with the total muscle volume. The similar R^2^ between the references we used and that of Shen and others with regard to L3 muscle area suggest that this may be a small limitation, but further validation is warranted. Another limitation is that we do not have any clinical data to correlate our findings, it is therefore difficult to set reference values as we do not know which patients actually suffer from sarcopenia. In addition, we did not visually inspect all the automatic segmentations, leaving a risk that there may be faulty segmentations that could distort the results. However, in the random 5% sample that was inspected no aberrant segmentations were detected, suggesting that the effect of faulty segmentations is likely small. Finally, a very large majority of the CT scans included in the study were made in men due to a large proportion of the patients had performed PET/CT scans for evaluation of prostate cancer. Analyzing females separately showed results very similar to the group as a whole, however, so this limitation seems to be small.

## 5 Conclusions

The skeletal muscle volumes Sacrum 25 cm, Sternum 25 cm and Sternum–Th12 are more accurate as proxies of total muscle volume than the single-slice areas commonly used, especially if combined with patient body height. These volumes can easily be measured through the cloud-based image analysis tool Recomia.org. Further studies are needed to define thresholds for clinical applications.

## CrediT author statement

**Thomas Ying**: Formal analysis, Data Curation, Writing - Original Draft, Writing - Review & Editing. **Pablo Borrelli**: Methodology, Validation, Writing - Review & Editing. **Lars Edenbrandt**: Conceptualization, Writing - Review & Editing, Funding acquisition. **Olof Enqvist**: Methodology, Software, Writing - Review & Editing. **Reza Kaboteh**: Resources, Writing - Review & Editing. **Elin Trägårdh**: Project administration, Funding acquisition, Writing - Review & Editing. **Johannes Ulén**: Methodology, Software, Formal analysis, Writing - Review & Editing. **Henrik Kjölhede**: Conceptualization, Formal analysis, Investigation, Supervision, Writing - Original Draft, Writing - Review & Editing.

## Conflicts of interest

The authors declare no competing interests. None of the components in **recomia. org** are patented. The platform is free to use for scientific purposes in collaboration with the foundation.
