# Supplementary material for: AI-based fully automatic image analysis: Optimal abdominal and thoracic segmentation volumes for estimating total muscle volume on computed tomography scans
Source: Osteoporos Sarcopenia. 2024 Apr 24;10(2):78–83. doi: 10.1016/j.afos.2024.04.001 (PMC11260007; doi:10.1016/j.afos.2024.04.001)
Supplement: Multimedia component 1 [file mmc1.docx]

Supplementary Table 1. Cross table of tests of statistical significance of difference in correlation coefficients for the different models. P-values were calculated according to the method described by Hittner et al. Models with height are bivariable (including body height) while the remaining are univariable.

|  | L3 | L3 with height | Sacrum - L1 | Sacrum - L1 with height | Sacrum 25 cm | Sacrum 25 cm with height | Sternum-Th12 | Sternum-Th12 with height | Sternum 25 cm | Sternum 25 cm with height | Th10-Th12 | Th10-Th12 with height | Th12 | Th12 with height |
| --- | --- | --- | --- | --- | --- | --- | --- | --- | --- | --- | --- | --- | --- | --- |
| L3 | - | <0.0001 | <0.0001 | <0.0001 | <0.0001 | <0.0001 | <0.0001 | <0.0001 | <0.0001 | <0.0001 | 0.0398 | 0.7734 | 0.0001 | 0.1773 |
| L3 with height | | - | <0.0001 | <0.0001 | <0.0001 | <0.0001 | 0.0002 | <0.0001 | 0.0058 | <0.0001 | <0.0001 | 0.0015 | <0.0001 | 0.0357 |
| Sacrum - L1 | |  | - | 0.0083 | <0.0001 | <0.0001 | 0.2583 | 0.0107 | 0.0351 | 0.0001 | <0.0001 | <0.0001 | <0.0001 | <0.0001 |
| Sacrum - L1 with height | | |  | - | <0.0001 | <0.0001 | 0.0953 | 0.0460 | 0.0079 | 0.0007 | <0.0001 | <0.0001 | <0.0001 | <0.0001 |
| Sacrum 25 cm | |  |  |  | - | <0.0001 | <0.0001 | 0.0025 | <0.0001 | 0.1153 | <0.0001 | <0.0001 | <0.0001 | <0.0001 |
| Sacrum 25 cm with height | | |  |  |  | - | <0.0001 | <0.0001 | <0.0001 | 0.0005 | <0.0001 | <0.0001 | <0.0001 | <0.0001 |
| Sternum-Th12 | |  |  |  |  |  | - | <0.0001 | 0.1635 | <0.0001 | <0.0001 | <0.0001 | <0.0001 | <0.0001 |
| Sternum-Th12 with height | | |  |  |  |  |  | - | <0.0001 | 0.0517 | <0.0001 | <0.0001 | <0.0001 | <0.0001 |
| Sternum 25 cm | |  |  |  |  |  |  |  | - | <0.0001 | <0.0001 | <0.0001 | <0.0001 | <0.0001 |
| Sternum 25 cm with height | | |  |  |  |  |  |  |  | - | <0.0001 | <0.0001 | <0.0001 | <0.0001 |
| Th10-Th12 | |  |  |  |  |  |  |  |  |  | - | <0.0001 | 0.0299 | <0.0001 |
| Th10-Th12 with height | | |  |  |  |  |  |  |  |  |  | - | <0.0001 | 0.1253 |
| Th12 |  |  |  |  |  |  |  |  |  |  |  |  | - | <0.0001 |
| Th12 with height | |  |  |  |  |  |  |  |  |  |  |  |  | - |

Supplementary Table 2. Summary of the regression models evaluated in the study. The models are described by the formula:

$volume=intercept+c1*value+c2*height+c3*value*height$, where the constants c are given in the table.

| Model | Intercept (95% CI) | Value (95% CI) | Height (95% CI) | Value * Height (95% CI) |
| --- | --- | --- | --- | --- |
| Sacrum 25 cm  + height | -1005 (-1189– -822)  -1024 (-5335–3286) | 2.4 (2.3–2.4)  1.2 (0.28–2.1) | -  391 (-2101–2883) | -  0.58 (0.05–1.1) |
| L3  + height | 321 (44–598)  -7420 (-13737– -1103) | 0.63 (0.61–0.64)  0.44 (0.03–0.85) | -  5079 (1436–8722) | -  0.06 (-0.17–0.29) |
| Sacrum–L1  + height | 455 (261–648)  -10151 (-14750– -5552) | 1.8 (1.8–1.8)  3.3 (2.5–4.2) | -  6339 (3686–8992) | -  -0.93 (-1.4– -0.45) |
| Th10 – Th12  + height | 2295 (2055–2534)  -13919 (-19331– -8507) | 6.6 (6.4–6.8)  13 (8.0–17) | -  9919 (6799–13038) | -  -4.0 (-6.6– -1.4) |
| Sternum – Th12  + height | 2479 (2311–2648)  -8238 (-11828– -4647) | 2.2 (2.2–2.3)  2.8 (1.8–3.9) | -  6604 (4534–8675) | -  -0.48 (-1.1–0.11) |
| Th12  +height | 1.653 (1366–1939)  -13208 (-19203– -7213) | 0.79 (0.76–0.81)  0.90 (0.34–1.5) | -  9203 (5749–12657) | -  -0.14 (-0.45–018) |
| Sternum 25 cm  + height | 1122 (924–1319)  -10182 (-14272– -6092) | 2.7 (2.7–2.8)  2.8 (1.6–4.1) | -  7073 (4721–9425) | -  -0.25 (-1.0–0.47) |

Supplementary Table 3. Performance characteristics of regression models for predicting total torso muscle volume from a limited volume or slice area for the patients with body height data available with respect to age, grouped by age quartiles (Q0-25, Q25-75, and Q75-100). The best model in each category is shown in bold.

| **Q0-25** |  |  |  |  | **Q25-75** |  |  |  |  | **Q75-100** |  |  |  |
| --- | --- | --- | --- | --- | --- | --- | --- | --- | --- | --- | --- | --- | --- |
|  | AIC | R^2^ | RMSE |  |  | AIC | R^2^ | RMSE |  |  | AIC | R^2^ | RMSE |
| **Abdomen** |  |  |  |  | **Abdomen** |  |  |  |  | **Abdomen** |  |  |  |
| Sacrum 25 cm | 2,039 | 0.942 | 681 |  | Sacrum 25 cm | 6,985 | 0.918 | 518 |  | Sacrum 25 cm | 3,039 | 0.915 | 475 |
| Sacrum 25 cm with height | **2,029** | **0.947** | **645** |  | Sacrum 25 cm with height | **6,923** | **0.928** | **482** |  | Sacrum 25 cm with height | **3,019** | **0.924** | **447** |
| Sacrum - L1 | 2,098 | 0.908 | 857 |  | Sacrum - L1 | 7,157 | 0.880 | 626 |  | Sacrum - L1 | 3,145 | 0.856 | 619 |
| Sacrum - L1 with height | 2,097 | 0.910 | 840 |  | Sacrum - L1 with height | 7,129 | 0.888 | 604 |  | Sacrum - L1 with height | 3,142 | 0.860 | 609 |
| L3 | 2,138 | 0.875 | 1,000 |  | L3 | 7,447 | 0.773 | 861 |  | L3 | 3,258 | 0.746 | 822 |
| L3 with height | 2,114 | 0.898 | 899 |  | L3 with height | 7,342 | 0.820 | 764 |  | L3 with height | 3,227 | 0.785 | 753 |
| **Thorax** |  |  |  |  | **Thorax** |  |  |  |  | **Thorax** |  |  |  |
| Sternum 25 cm | 2,107 | 0.902 | 887 |  | Sternum 25 cm | 7,257 | 0.850 | 698 |  | Sternum 25 cm | 3,172 | 0.836 | 662 |
| Sternum 25 cm with height | 2,057 | 0.934 | 719 |  | Sternum 25 cm with height | **7,062** | **0.903** | **562** |  | Sternum 25 cm with height | **3,099** | **0.887** | **546** |
| Sternum-Th12 | 2,077 | 0.922 | 788 |  | Sternum-Th12 | 7,202 | 0.867 | 657 |  | Sternum-Th12 | 3,197 | 0.814 | 705 |
| Sternum-Th12 with height | **2,041** | **0.942** | **674** |  | Sternum-Th12 with height | 7,104 | 0.894 | 588 |  | Sternum-Th12 with height | 3,139 | 0.862 | 604 |
| Th10-Th12 | 2,138 | 0.875 | 1,001 |  | Th10-Th12 | 7,475 | 0.758 | 888 |  | Th10-Th12 | 3,315 | 0.664 | 946 |
| Th10-Th12 with height | 2,107 | 0.903 | 875 |  | Th10-Th12 with height | 7,418 | 0.788 | 830 |  | Th10-Th12 with height | 3,275 | 0.727 | 849 |
| Th12 | 2,183 | 0.822 | 1,193 |  | Th12 | 7,500 | 0.745 | 912 |  | Th12 | 3,309 | 0.674 | 933 |
| Th12 with height | 2,121 | 0.892 | 924 |  | Th12 with height | 7,365 | 0.811 | 783 |  | Th12 with height | 3,252 | 0.757 | 802 |
